# Diversity Patterns and a New Species of *Dendrocalamus* (Poaceae, Bambusoideae) in Yunnan, China

**DOI:** 10.3390/plants14213364

**Published:** 2025-11-03

**Authors:** Jianwei Li, Maosheng Sun, Wanling Qin, Haofeng Bao, Chaomao Hui, Weiyi Liu

**Affiliations:** 1 Institute of Bamboo and Rattan, Sympodium Bamboo Engineering Technology Research Center, College of Forestry, Southwest Forestry University, Kunming 650224, China; ynjianweili@126.com (J.L.); maoshs@imbcams.com.cn (M.S.); bhfzmx@163.com (H.B.); 2 Guangnan Babao Provincial Nature Reserve Administration Bureau, Wenshan 663399, China; qwl0202@139.com

**Keywords:** Yunnan Province, *Dendrocalamus*, diversity, geographical distribution, new species, phylogenetic analysis

## Abstract

To systematically investigate the diversity and distribution patterns of *Dendrocalamus* in Yunnan Province, we integrated field surveys, literature reviews, specimen records, and existing research data to compile and analyze the distribution of *Dendrocalamus* species across the region. The results revealed the following: (1) A total of 3730 valid distribution points were compiled, representing 38 taxa of *Dendrocalamus* (including 32 species, 3 varieties, and 3 forms), reflecting remarkably high species diversity. These account for approximately 52% (38/73) of the global species and 69% (38/55) of those recorded in China. (2) Horizontal Distribution Pattern: In terms of distribution points, Pu’er had the highest count (929), followed by Xishuangbanna (759) and Lincang (586). Honghe, Wenshan, and Dehong also showed substantial records. Regarding species richness, Xishuangbanna ranked highest with over 20 species, while Pu’er and Honghe contained 15–20 species. Yuxi and Kunming supported 10–15 species, and Baoshan, Nujiang, Chuxiong, Wenshan, Qujing, and Zhaotong each hosted 5–10 species. In contrast, Dali, Lijiang, and Diqing recorded only 0–5 species. (3) Vertical Distribution Pattern: Distribution points were predominantly concentrated in the 1000–1500 m elevation range, whereas species richness peaked in the 500–1000 m band. Both the number of distribution points and species richness were lowest at elevations above 2500 m. (4) Based on the collected 3730 distribution points, kernel density analysis and hot spot analysis (Getis-Ord Gi*) were performed in ArcGIS 10.8. Both analyses indicated that southern Yunnan (centered on Xishuangbanna and Pu’er) exhibits significant spatial clustering characteristics, identifying it as the core distribution area for *Dendrocalamus* species in Yunnan Province. (5) During field surveys, a distinctive new species characterized by swollen internodes was discovered. Morphological comparison and phylogenetic analysis confirmed it as a new species of *Dendrocalamus* and named *Dendrocalamus turgidinodis* C.M.Hui, M.S.Sun & J.W.Li, it is similar to *D. hamiltonii*, *D. fugongensis*, and *D. sinicus*, but can be easily distinguished by culm diameter 13–16 cm, intranode swollen, culm leaf sheath deciduous, culm blade erect, culm leaf ligule ca. 5 mm tall., Foliage leaf ligule 1–1.5 mm tall (vs. 1.5–2 mm). In conclusion, this study demonstrates that Yunnan Province serves as a major distribution center for *Dendrocalamus*, with the genus primarily distributed from the southeastern to southwestern parts of the region, and concentrated most densely in the southern area encompassing Xishuangbanna and Pu’er.

## 1. Introduction

Yunnan Province, situated in southwestern China (21°08′–29°15′ N, 97°39′–106°12′ E) with an elevation range of 76.4–6740 m, borders Vietnam, Laos, and Myanmar. It lies at the convergence of three distinct physiographic regions: the East Asian tropical monsoon zone, the Indochina Peninsula tropical monsoon zone, and the Qinghai–Tibet Plateau [1]. The region is characterized by major rivers such as the Jinsha, Lancang, and Nujiang, and mountain ranges including the Meri Snow Mountain, Gaoligong Mountains, and Ailao Mountains. This complex and diverse topography has provided exceptional conditions for the concentration, dispersal, and differentiation of bamboo plants, making Yunnan one of the world’s richest regions in bamboo species diversity and variety of ecological types and natural bamboo forests, earning it the reputation as the “Hometown of Bamboos” [2,3]. According to recent statistics, Yunnan hosts 41 genera and 389 species of bamboos (including cultivated taxa) [4]. In recent years, numerous new species within the bamboo subfamily (Bambusoideae) continue to be described from Yunnan [5,6,7,8,9,10,11,12,13,14,15,16,17,18,19].

The unique natural environment of Yunnan highly favors the growth and differentiation of bamboo plants, making it one of the centers of bamboo origin and distribution. It has nurtured several bamboo genera with Yunnan as their distribution center, among which the genus *Dendrocalamus* Nees, 1835 is a typical representative [2,3,20]. *Dendrocalamus* was established by the German scholar Nees von Esenbeck in 1834 [21]. It belongs to the subtribe Bambusinae (Poaceae, Bambusoideae) and comprises paleotropical woody bamboos, primarily distributed in tropical and subtropical regions of Asia, including India, China, Indonesia, Sikkim, Myanmar, Vietnam, and Laos. Key diagnostic characteristics of *Dendrocalamus* include: caespitose culms, erect with nodding apex; internodes terete, culm walls thick, basal internodes nearly solid; prominent sheath scars; flat nodes; intranodes usually with dense tomenta; multiple branches per node, with dominant branches conspicuous or not; culm leaves deciduous, leathery; auricles inconspicuous or absent; ligules conspicuous; blades reflexed; numerous leaves per branchlet; auricles absent or inconspicuous; ligules developed; blades large [22,23,24,25]. Globally, *Dendrocalamus* comprises approximately 66 species [26]. China records 38 species, 3 varieties, 8 forms, and 1 hybrid, mainly distributed in the southern and southwestern parts of the country. Yunnan, being the core distribution area, harbors 27 species, 1 variety, and 3 forms [24,25]. Notably, new species of *Dendrocalamus* continue to be described from Yunnan in recent years [8,13,16].

Despite Yunnan being a distribution center for *Dendrocalamus*, the precise number of species occurring within the province and their distribution patterns remain inadequately documented. To address this, the present study integrated field investigations (2023–2025), herbarium specimen consultations (via the China Virtual Herbarium (CVH), the National Specimen Information Infrastructure (NSII)), literature reviews (including monographs and newly published species), and historical survey records from the research team. This comprehensive approach aimed to clarify the species composition and geographical distribution patterns of *Dendrocalamus* within Yunnan, thereby establishing a crucial data foundation for in-depth research on this genus and the overall bamboo diversity of the region.

Furthermore, during field investigations, the authors discovered a clump of an unknown bamboo species. It exhibits a slightly curved culm tip; multiple branches with 1–3 dominant ones; absence of culm leaf auricles; conspicuous culm leaf ligules; and erect culm leaf sheath blades—morphological characteristics consistent with the defining features of *Dendrocalamus*. Phylogenetic analysis based on molecular data also confirms its placement within *Dendrocalamus*. This species is formally described herein as new to science.

## 2. Materials and Methods

### 2.1. Field Investigation

Primary investigation areas were determined based on the known distribution patterns of *Dendrocalamus* species and the research team’s prior survey experience. During field work, key habitat factors such as GPS coordinates, altitude, slope, and aspect were systematically recorded for each distribution site. Detailed observations were made on key diagnostic characteristics, including clump morphology, culm features (diameter at breast height, height, and age structure), branching pattern, culm sheath morphology, and leaf characteristics. Information on local uses and vernacular names was also documented. Voucher specimens comprising culm sheaths, culm segments, branches, leaves, and flowers or fruits (when available) were collected for further laboratory identification.

### 2.2. Consultation of Primary Records

The species composition and geographical distribution of *Dendrocalamus* in Yunnan Province were synthesized and organized through systematic consultation of authoritative floras and related literature, including *Flora Reipublicae Popularis Sinicae* [22], *Flora of China* [23], *Iconographia Bambusoidearum Sinicarum* [24], *Illustrated Flora of Bambusoideae in China* [25], *Flora Yunnanica* [27], and *Yunnan Bamboo Flora* [3]. This was supplemented by examining herbarium specimens housed in the China Virtual Herbarium (CVH), the National Specimen Information Infrastructure (NSII), the Herbarium of Kunming Institute of Botany, Chinese Academy of Sciences (KUN), and the Herbarium of Southwest Forestry University (SWFC), as well as integrating historical survey data from the research team.

### 2.3. Specimen Identification

Morphological observations of the collected voucher specimens were conducted using a stereomicroscope (ASOV HD201, AOSVI Optical Instrument Co., Ltd., Shenzhen, China). Identification was performed following the taxonomic treatments in *Flora Reipublicae Popularis Sinicae* [22], *Flora of China* [23], *Iconographia Bambusoidearum Sinicarum* [24], *Illustrated Flora of Bambusoideae in China* [25], *Flora Yunnanica* [27], and *Yunnan Bamboo Flora* [3]. All identification data, cross-referenced with original field records, were systematically compiled into a Microsoft Excel spreadsheet.

### 2.4. Verification of Bamboo Species Names

This study is limited to naturally distributed species. Species names were verified and corrected according to http://www.theplantlist.org (accessed on 19 May 2025) and https://powo.science.kew.org/ (accessed on 19 May 2025), excluding cultivated and introduced taxa. The final nomenclature for all species followed the standards of *Flora of China* [23] and *Flora Reipublicae Popularis Sinicae* [22].

### 2.5. Collation and Vectorization of Distribution Points

We integrated field survey records, specialized monographs, literature, specimen information, and historical data from the research group to systematically collate the distribution points of *Dendrocalamus* plants in Yunnan Province. Location information was verified and determined according to the following principles: (1) If original records (field records, specimen labels, or literature) included latitude, longitude, and altitude information, these data were directly adopted. (2) If original records lacked coordinates but provided specific minor place names, high-resolution remote sensing images (e.g., MapWorld) were used to precisely locate their geographic coordinates, with corresponding altitude values extracted using Yunnan Province’s digital elevation model (DEM) data. (3) Records lacking reliable location information were excluded. All verified distribution point information was recorded in an Excel spreadsheet, and a point vector layer was generated in ArcGIS 10.8 [28] for subsequent spatial analysis.

### 2.6. Data Gridding

**Figure 1:**
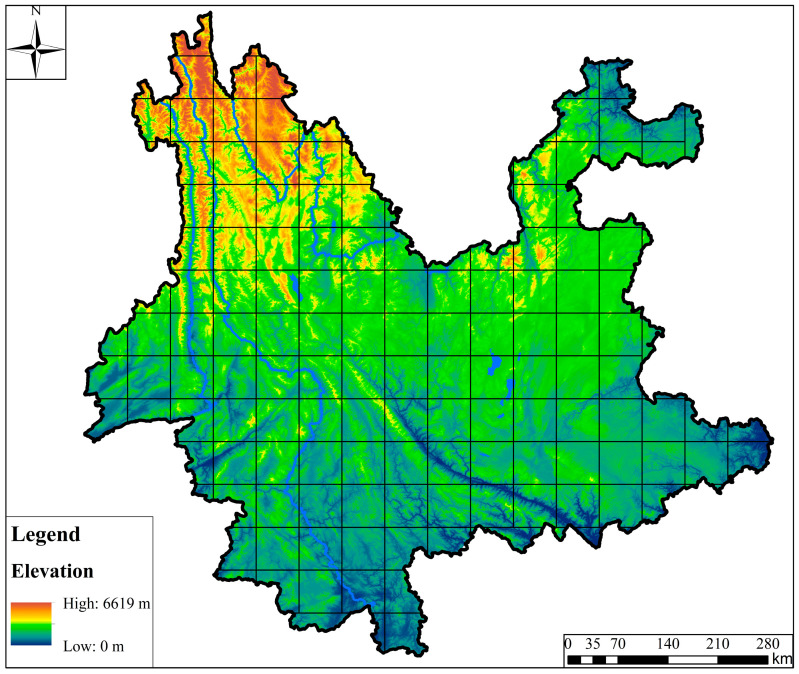
Distribution of grids in Yunnan.

To analyze the macro-scale distribution patterns of *Dendrocalamus* in Yunnan Province, we employed a spatial grid sampling approach. Considering that some species occurrence records were only accurate to the county administrative level, we selected a grid size of 60 km × 60 km. Grid cells with less than 50% of their area within the boundary of Yunnan Province were excluded, resulting in a final set of 103 grid cells for analysis (Figure 1). This grid scale is slightly larger than the average area of a county in Yunnan Province (approximately 3056 km^2^), ensuring that each grid cell functions as an independent macro-geographic analytical unit, effectively integrating distribution information at the county level and reducing bias introduced by locational uncertainty.

Furthermore, using 30 m resolution ASTER Global Digital Elevation Model data (https://www.gscloud.cn/), we extracted altitude data for each species occurrence point within the grids, thereby elucidating the distribution patterns of *Dendrocalamus* species across Yunnan Province.

### 2.7. Geographical Distribution Patterns

Plant species distribution patterns are influenced by various environmental factors (e.g., precipitation, temperature, environmental heterogeneity), which often exhibit regular variations along longitudinal, latitudinal, or altitudinal gradients, leading to specific distribution patterns across spatial scales [29].

This study analyzes the distribution patterns of *Dendrocalamus* in Yunnan Province from both horizontal and vertical dimensions:

(1) Horizontal distribution pattern: Using administrative divisions (16 prefectures/cities) as analysis units, spatial distribution characteristics along longitudinal and latitudinal gradients are examined.
(2) Vertical distribution pattern: Employing altitudinal gradient analysis, elevation zones are divided at 500 m intervals, resulting in six zones: <500 m, 500–1000 m, 1000–1500 m, 1500–2000 m, 2000–2500 m, and >2500 m.

### 2.8. Kernel Density and Hot Spot Analysis

Kernel density analysis estimates the spatial density distribution of point features per unit area using kernel functions, without requiring a preset data distribution model. This method fully utilizes the spatial information of the original data, objectively characterizing the continuous variation in species’ spatial distribution and visually displaying areas of high and low species density [30].

Hot spot analysis identifies spatial clustering patterns by calculating the local spatial statistic Gi* value for each feature. It computes the weighted sum of the target feature and its neighbors (with weights decaying with distance), compares it with the expected global sum, and outputs standardized z-scores and corresponding p-values to determine spatial clustering patterns [31].

The processed vector data of *Dendrocalamus* species distribution points were imported into ArcGIS 10.8, and kernel density analysis and Getis-Ord Gi* hot spot analysis were applied. Kernel density analysis revealed continuous density patterns of *Dendrocalamus* distribution, while hot spot analysis identified statistically significant hot spots, cold spots, and non-significant areas, comprehensively analyzing spatial distribution characteristics.

### 2.9. DNA Extraction, Sequencing, and Assembly

The whole-genome DNA of the new *Dendrocalamus* species was isolated from silica gel-dried leaves using the TIANGEN Magnetic Plant Genomic DNA Kit (TIANGEN, Beijing, China), strictly following the kit’s protocol. The quality of the whole-genome DNA samples was first assessed for degradation, contamination, and concentration. Qualified genomic DNA samples were sheared into fragments of approximately 350 bp using a Covaris LE220R-plus (Covaris, Woburn, MA, USA). The DNA fragments were then subjected to end repair, A-tailing, and ligation with full-length adapters for Illumina sequencing, followed by further PCR amplification. The PCR products were accurately quantified for effective library concentration using real-time PCR (3 nM). DNA libraries meeting the standards were sequenced on the Illumina platform with PE150, generating approximately 2 GB of raw data per sample. All sequencing experiments described above were conducted at Novogene Bioinformatics Technology Co., Ltd. in Beijing, China.

After obtaining the raw data in FASTQ format, Fastp 0.19.7 [32] was used for quality control to filter out low-quality sequences. The chloroplast genome sequences were assembled using GetOrganelle 1.7.7.1 [33] with default parameters. The assembled sequences were then visualized and checked for circularization using Bandage 0.8.1 [34]. Annotation of the chloroplast genome was performed on CPGAVAS2 [35] based on the annotation information of *Dendrocalamus strictus* (Roxb.) Nees (NCBI NC_050776.1), followed by manual adjustments in Geneious Prime 2025.1.2 [36] to obtain the complete chloroplast genome of the target species.

### 2.10. Construct Phylogenetic Tree

As shown in previous studies [15,17,19], constructing phylogenetic trees using chloroplast genomes has proven to be a reliable and effective method for identifying new bamboo species. Therefore, in this study, we collected 40 chloroplast genome sequences from related species (Table 1). Chloroplast sequences of all species were aligned using MAFFT 7.525 [37]. Maximum likelihood (ML) analysis was performed using IQ-TREE 2.2.5 [38] with 1000 ultrafast bootstrap replicates and the SH-aLRT test [39]. Bayesian inference (BI) was conducted using MrBayes 3.2.7a [40], with the GTR+I+G model selected by BIC in jModelTest 2.1.7 [41]. The Markov Chain Monte Carlo (MCMC) simulation was run for 1,000,000 generations, sampling every 1000 generations, and the first 25% of iterations were discarded as burn-in. A 50% majority-rule consensus tree was constructed when the average standard deviation of split frequencies fell below 0.01. Posterior probabilities for each branch were calculated to assess support [40].

## 3. Results

### 3.1. Diversity Composition

Based on integrated field survey data, literature records, specimen verification, and historical research data, this study systematically determined the diversity of *Dendrocalamus* species in Yunnan Province. The results indicate that a total of 38 species (including the new species described herein) of *Dendrocalamus* are distributed in Yunnan, comprising 32 species, 3 varieties, and 3 forms, reflecting extremely high species diversity (Table 2).

### 3.2. Characteristics of Geographical Distribution Patterns

#### 3.2.1. Horizontal Distribution Pattern

A total of 3730 valid distribution points of *Dendrocalamus* in Yunnan Province were collected. Pu’er had the highest number of distribution points (929), followed by Xishuangbanna (759) and Lincang (586). Considerable numbers of distribution points were also recorded in Honghe, Wenshan, and Dehong, while no distribution points were observed in Diqing (Figure 1).

**Figure 2:**
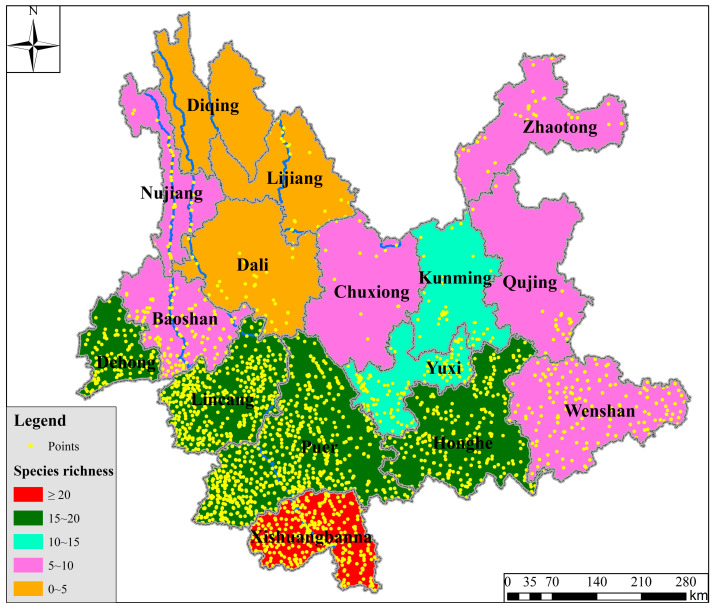
Horizontal Distribution Patterns of *Dendrocalamus* Diversity in Yunnan Province.

Species richness also exhibited significant regional variation. Xishuangbanna had over 20 species; Pu’er and Honghe contained 15–20 species; Yuxi and Kunming supported 10–15 species (notably, Kunming’s records primarily consist of species introduced from other regions in Yunnan, but due to their long-term establishment, they are included here). Baoshan, Nujiang, Chuxiong, Wenshan, Qujing, and Zhaotong hosted 5–10 species each. In contrast, Dali, Lijiang, and Diqing recorded only 0–5 species (Figure 2).

In summary, the core distribution area of *Dendrocalamus* in Yunnan Province is concentrated in a belt stretching from southwestern Yunnan (Lincang and Pu’er), through southern Yunnan (Xishuangbanna), to southeastern Yunnan (Honghe), forming an arc-shaped distribution belt along the southwestern border.

#### 3.2.2. Vertical Distribution Pattern

**Figure 3:**
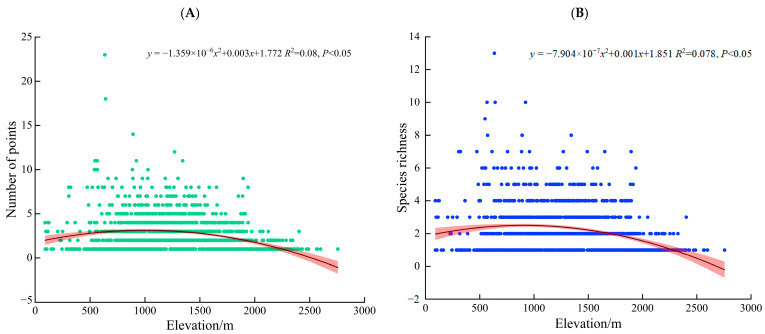
Vertical Distribution Patterns of *Dendrocalamus* in Yunnan Province. (**A**) Vertical distribution curve of occurrence points; (**B**) Vertical distribution curve of species richness.

Analysis of the altitudinal gradient based on the 3730 distribution points revealed that the number of distribution points is predominantly concentrated in the 1000–1500 m elevation band. Species richness peaks in the 500–1000 m elevation band. In contrast, both the number of distribution points and species richness are lowest at elevations above 2500 m (Figure 3).

#### 3.2.3. Spatial Patterns Revealed by Kernel Density and Hot Spot Analysis

**Figure 4:**
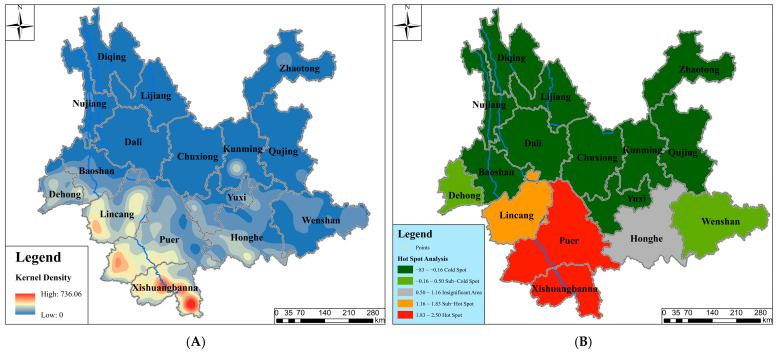
Comparison of Kernel Density and Hot Spot Analysis. (**A**) Kernel Density; (**B**) Hot Spot Analysis.

Based on the 3730 distribution points, kernel density analysis and hot spot analysis (Getis-Ord Gi*) were performed in ArcGIS 10.8 (Figure 4). Both analyses indicate that southern Yunnan (centered on Xishuangbanna Prefecture) exhibits significant spatial clustering characteristics, identifying it as the core distribution area for *Dendrocalamus* in Yunnan Province. Furthermore, Pu’er City also constitutes an important distribution region.

### 3.3. A New Species, Dendrocalamus turgidinodis C.M.Hui, M.S.Sun & J.W.Li, sp.nov.

#### 3.3.1. Morphological Characteristics Analysis

Through comparison of morphological characteristics, we found that *Dendrocalamus turgidinodis* is similar to *D. hamiltonii*, *D. fugongensis*, and *D. sinicus*, but it can be distinguished by several key morphological features, such as the structures of the intranode, culm leaf ligule, and culm blade (Table 3).

#### 3.3.2. Taxonomic Treatment

**Figure 5:**
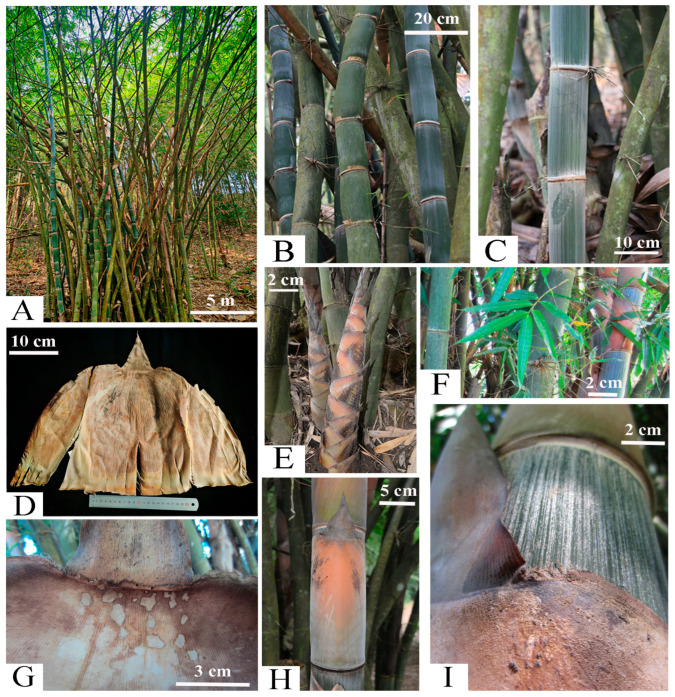
*Dendrocalamus turgidinodis* C.M.Hui, M.S.Sun & J.W.Li. (**A**) Habit; (**B**) Culm swollen; (**C**) Young culm; (**D**) Culm leaf; (**E**) New shoot; (**F**) Fresh foliage leaves; (**G**) Culm leaf ligule; (**H**) Culm blade; (**I**) Base of culm blade.

**Figure 6:**
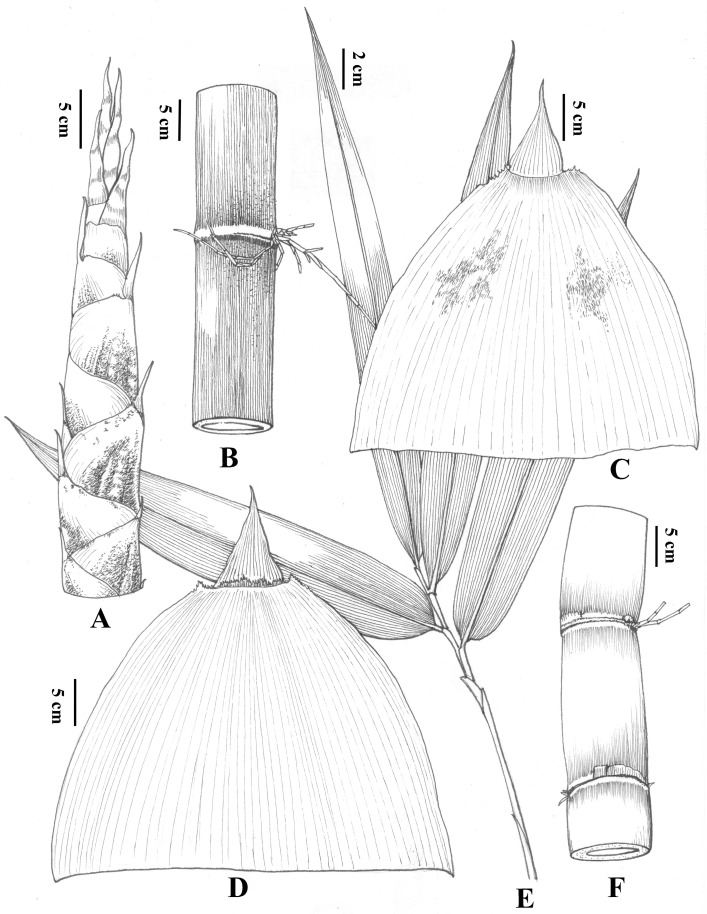
*Dendrocalamus turgidinodis* C.M.Hui, M.S.Sun & J.W.Li. (**A**) New shoot; (**B**) Young culm and branches; (**C**) Culm leaf, adaxial view; (**D**) Culm leaf, abaxial view; (**E**) Fresh foliage leaves; (**F**) Culm swollen.

***Dendrocalamus turgidinodis*** C.M.Hui, M.S.Sun & J.W.Li, sp.nov. Figure 5 and Figure 6.

**Chinese name:** “gǔ jié lóng zhú” (鼓节龙竹, referring to a *Dendrocalamus* species characterized by swollen internodes); “wǎ pǔ” (local Lahu language).

**Local use:** The local Lahu people call it “wǎ pǔ,” which refers to its characteristic of internode swollen. Its bamboo shoots are also used to make sour shoots.

**Type.** Papeng Village, Menglai Township, Cangyuan County, Lincang City, Yunnan Province, China, in sparse forests, alt. 1365 m, 23°11′54.31″N, 99°14′50.58″E, 24 September 2024, *C. M. Hui, J. W. Li, H. F. Bao & C. H. Zhang* 0072414 (holotype: SWFC!), 0072415 (isotype: SWFC!).

**Diagnosis.** *Dendrocalamus turgidinodis* is similar to *D. hamiltonii*, *D. fugongensis*, and *D. sinicus*, but can be easily distinguished by culm diameter 13–16 cm (vs. 9–13 cm in *D. hamiltonii* and 20–30 cm in *D. sinicus*), intranode swollen (vs. not in *D. hamiltonii* and *D. fugongensis*), culm leaf sheath deciduous (vs. tardily deciduous or persistent in *D. sinicus*), culm blade erect (vs. nearly erect or a little open in *D. sinicus*), culm leaf ligule ca. 5 mm tall (vs. ca. 1 mm in *D. hamiltonii*, ca. 3 mm in *D. fugongensis* and ca. 6 mm in *D. sinicus*), Foliage leaf ligule 1–1.5 mm tall (vs. 1.5–2 mm) (Table 3).

**Description:** Rhizomes pachymorph. Culms erect, apex nodding, 17–25 m tall, 10–16 cm diameter, basal nodes with aerial roots; internodes 25–34 (40) cm long, conspicuously swollen, and covered with white longitudinal tomentum on the surface when young; intranode 3–5 mm tall, with a ring of white to yellowish-brown tomentum; culm nodes slightly prominent with densely white tomentum; sheath scar prominent, with a ring of brown setae. Branching from the first or second node, multiple branches per node with 1–3 dominant, several slender branchlets curve backward and encircle the culm node. Culm leaves leathery, deciduous, white powder and deciduous black setae abaxially, 27–30 cm long, 29–33 cm wide, middle-upper portion with ca. 3–5 mm wide thin membranous margin with sparsely cilia, two shoulders of sheaths a little prominent with margin serrate; auricles absent; ligule ca. 5 mm tall, margin dentate with short oral setae; blades erect, 10–13 cm long, ca. 4/5 as long as the apex of sheaths, wrinkled, margin involute. Foliage leaves 6–10 per ultimate branch; blades 11–33 cm × 1.5–8 cm, glabrous, secondary veins 6–10 pairs, transverse veins inconspicuous; petiole ca. 5 mm long, glabrous; sheaths 1.5–2.2 cm long, with white powder and margin sparsely ciliate; auricles absent; ligule 1–1.5 mm tall, margin serrate. Inflorescence and caryopsis unknown.

#### 3.3.3. Phylogenetic Analysis

**Figure 7:**
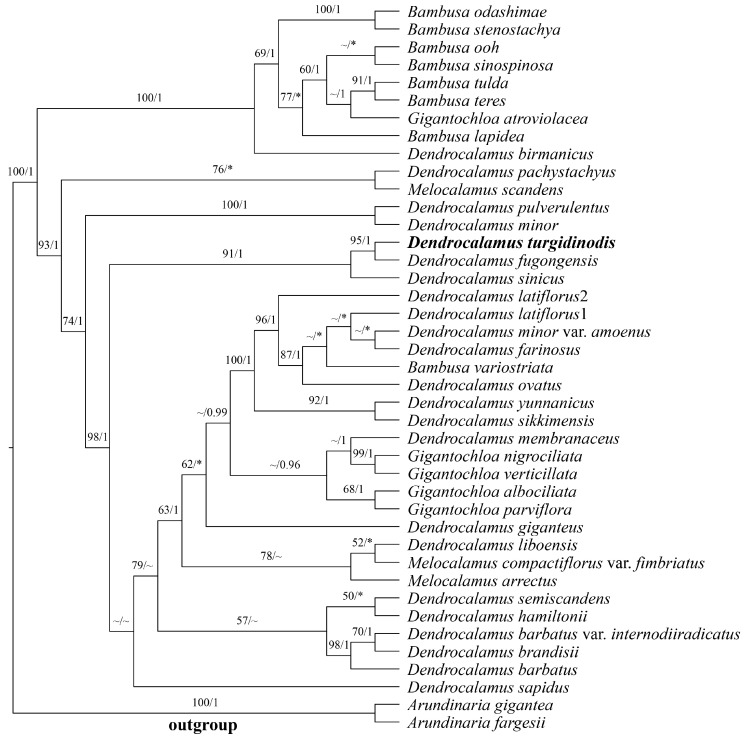
Phylogenetic tree reconstructed by Maximum Likelihood (ML) and Bayesian Inference (BI) analysis based on plastome sequences. Numbers along branches indicate the Maximum Likelihood bootstrap values (MLBP) (**left**) and Bayesian posterior probabilities (BI) (**right**). “~”: nodes with Maximum Likelihood bootstrap values (MLBP) <50% (**left**), Bayesian posterior probabilities (BI) <95% (**right**). *: inconsistent topological structures.

The total length of the plastome sequence was 159,118 bp, including 7196 variable sites and 1684 parsimony-informative sites. In the plastome phylogenetic tree, the new species formed a clade with other *Dendrocalamus* species (MLBP/BI = 91/1) and was most closely related to *D. fugongensis* and *D. sinicus* (Figure 7).

**Phenology:** Shooting from August to October.

**Etymology:** The specific epithet “*turgidinodis*” is derived from the distinctive morphological feature of its swollen internodes.

**Distribution and habitat:** *Dendrocalamus turgidinodis* is endemic to Yunnan, China, and is currently found only near village at an elevation of 1376 m in Cangyuan County.

**Conservation status:** Although relatively detailed surveys have been conducted around the type locality, many similar habitats in other regions remain unsurveyed. As conditions in these areas are unclear, we recommend classifying it as Data Deficient (DD) following the IUCN categories and criteria [42].

**Additional specimens examined.** CHINA. Yunnan: Lincang City, Cangyuan County, Menglai Township, Papeng Village, 23°11′54.31″ N, 99°14′50.58″ E, 1376 m alt., 24 September 2024, *C. M. Hui, J. W. Li, H. F. Bao & C. H. Zhang* 0072416 (SWFC!).

## 4. Discussion

### 4.1. Diversity Composition Analysis

Bamboo species originated in tropical and subtropical rainy regions. Yunnan Province, under the dual influence of southeast and southwest monsoons, combined with its complex topography and significant elevation variations, has formed diverse habitats. This environmental context is not only suitable for the widespread growth of bamboo plants but also provides ideal conditions for species differentiation.

Through approximately two years of systematic data collection and field surveys, this study obtained 3730 valid distribution points of *Dendrocalamus* in Yunnan Province, encompassing 38 species. To date, approximately 73 species of *Dendrocalamus* are known globally, with about 55 species distributed in China. Based on these figures, the number of *Dendrocalamus* species in Yunnan accounts for about 52% of the global total and approximately 69% of the Chinese total. Evidently, the proportion of *Dendrocalamus* species in Yunnan exceeds half of both global and national totals, demonstrating that Yunnan is one of the modern distribution centers of *Dendrocalamus*.

According to *Illustrated Flora of Bambusoideae in China* [25], Yunnan hosts 27 species, 1 variety, and 3 forms of *Dendrocalamus*. In contrast, this study documents 32 species, 3 varieties, and 3 forms. This discrepancy arises from the inclusion of three recently published new species [8,13,16] and the new species described herein. Additionally, we acknowledge *D. menglongensis* [43], *D. sinicus* var. *pachyloenus* [44], and *D. membranaceus* var. *microphyllum* (Chinese Virtual Herbarium specimen: HITBC 0024146). Consequently, the number of *Dendrocalamus* species recorded in this study is significantly higher than that in the *Illustrated Flora of Bambusoideae in China* [25].

### 4.2. Attributes of the Spatial Distribution Pattern

As a predominantly mountainous province, Yunnan’s complex topography and diverse drainage systems collectively create highly heterogeneous habitats. Spatial analysis of 3730 distribution records representing 38 *Dendrocalamus* species reveals pronounced horizontal distribution concentration in southern Yunnan, followed by southwestern and southeastern regions, while central to northwestern and northeastern areas show remarkably sparse distributions. Vertical distribution patterns demonstrate peak record occurrence within the 1000–1500 m elevation zone, whereas maximum species richness appears in the 500–1000 m altitudinal band.

These findings indicate that areas below 1500 m elevation in southeastern–southern–southwestern Yunnan constitute the core distribution range for *Dendrocalamus* in the province. This pattern corresponds strongly with the ecological characteristics of paleotropical woody bamboos. As a genus endemic to tropical–subtropical regions, *Dendrocalamus* depends on high temperature, humidity, and abundant rainfall, exhibiting a relatively narrow ecological adaptation range [45]. The southeastern–southern–southwestern Yunnan region, situated south of the Tropic of Cancer at the northern margin of the paleotropical flora, possesses multiple distinctive features: (1) It represents a transitional zone between Sino-Himalayan and Malaysian floras, directly adjacent to the Indochinese Peninsula; (2) Under the dual influence of Indian and Pacific Ocean monsoons, combined with the cold-air barrier effect of the northern Yunling-Hengduan Mountains, it develops a typical tropical monsoon climate characterized by sufficient sunlight and synchronous rainfall-heat patterns; (3) As the province’s low-latitude area, it maintains well-preserved tropical rainforest ecosystems. These characteristics collectively establish an ideal environment for the survival and differentiation of *Dendrocalamus* species in Yunnan Province.

### 4.3. Taxonomic Placement of the New Species

Based on comparative morphological characteristics and phylogenetic tree construction, it has been sufficiently demonstrated that the new species described in this study represents a previously unrecorded species within the genus *Dendrocalamus*. According to the classification system of *Flora Reipublicae Popularis Sinicae* [22], the genus *Dendrocalamus* can be divided into two subgenera, subgen. *Dendrocalamus* and subgen. *Sinocalamus*. The typical characteristics of the former include: culm tip only slightly curved; branching from lower nodes, 3 dominant branches; sheath leaf auricles usually absent; foliage leaf blades usually narrow. The diagnostic features of the latter include: culm tip filiform pendulous; branching from upper nodes, dominant branches inconspicuous or solitary; sheath leaf auricles small or reduced; foliage leaf blades broad. The morphological characteristics of this new species are as follows: branching from first or second nodes, 1–3 dominant branches, and sheath leaf auricles absent. Therefore, we assign it to subgen. *Dendrocalamus*. Furthermore, it is noteworthy that the unique characteristic of swollen internodes in this new species adds to the morphological diversity of the genus *Dendrocalamus*.

In this study, the phylogenetic tree from plastome sequence revealed that the new species clusters with *D. fugongensis* and *D. sinicus* into a single clade with high support (Figure 7), providing key evidence for determining its taxonomic status. The use of chloroplast genomes to delineate new bamboo species has been widely adopted in previous studies [15,17,19]. However, our phylogenetic tree shows that species of the *Dendrocalamus* do not form a monophyletic clade, a phenomenon closely related to the “BDG complex.” This complex, primarily composed of genera such as *Bambusa*, *Dendrocalamus*, and *Gigantochloa*, represents the most diverse and phylogenetically intricate group within the Bambusoideae subfamily. The delimitation of genera and their evolutionary relationships within this complex have long been contentious [45,46,47]. Thus, the polyphyletic distribution of *Dendrocalamus* species observed in this study reflects the complex evolutionary history of the BDG complex.

## 5. Conclusions

Our integrated findings robustly identify southern Yunnan, with Xishuangbanna and Pu’er at its core, as a critical diversity center and evolutionary hotspot for the genus *Dendrocalamus*. By synthesizing large-scale distribution data, spatial distribution patterns, and detailed morphological examination, we have not only systematically delineated a core distribution area harboring 38 taxa (32 species, 3 varieties, 3 forms) but also discovered and described a distinctive new species, *Dendrocalamus turgidinodis*, characterized by its swollen internodes.

The distinct horizontal and vertical distribution patterns, heavily concentrated in the sub-montane areas below 1500 m elevation, strongly correlate with the region’s tropical monsoon climate and complex topography. These environmental factors have collectively created a mosaic of niches that have likely driven both the preservation and in situ diversification of this paleotropical bamboo lineage.

The discovery of *D. turgidinodis* further enriches the morphological diversity of the genus and serves as a potent reminder of the incompletely documented biodiversity within this biogeographically crucial region. Consequently, this study underscores the paramount importance of southern Yunnan as a priority zone for both the continued scientific exploration of woody bamboos and the development of targeted conservation strategies.

## Figures and Tables

**Table 1:** Voucher information and GenBank accession numbers for plant materials used in this study.

Number	Taxon	GenBank Accession No.
1	*Bambusa lapidea*	NC059750
2	*Bambusa odashimae*	OM001092
3	*Bambusa ooh*	NC081017
4	*Bambusa sinospinosa*	NC050781
5	*Bambusa stenostachya*	NC063134
6	*Bambusa teres*	NC050751
7	*Bambusa tulda*	NC056166
8	*Bambusa variostriata*	MN038143
9	*Dendrocalamus barbatus*	NC050747
10	*Dendrocalamus barbatus* var. *internodiiradicatus*	MK679778
11	*Dendrocalamus birmanicus*	NC050750
12	*Dendrocalamus farinosus*	MZ681865
13	*Dendrocalamus fugongensis*	NC050764
14	*Dendrocalamus giganteus*	NC050752
15	*Dendrocalamus hamiltonii*	NC050746
16	*Dendrocalamus latiflorus*1	NC013088
17	*Dendrocalamus latiflorus*2	MW054656
18	*Dendrocalamus liboensis*	NC081047
19	*Dendrocalamus membranaceus*	NC050766
20	*Dendrocalamus minor*	NC050755
21	*Dendrocalamus minor* var. *amoenus*	MK679791
22	*Dendrocalamus ovatus*	NC050759
23	*Dendrocalamus pachystachyus*	NC050753
24	*Dendrocalamus pulverulentus*	NC050758
25	*Dendrocalamus sapidus*	NC050757
26	*Dendrocalamus semiscandens*	NC050748
27	*Dendrocalamus sikkimensis*	NC050760
28	*Dendrocalamus sinicus*	MK679768
29	*Dendrocalamus turgidinodis*	submission ID: 3013080
30	*Dendrocalamus yunnanicus*	MN782326
31	*Gigantochloa albociliata*	NC050765
32	*Gigantochloa atroviolacea*	NC050777
33	*Gigantochloa nigrociliata*	NC050778
34	*Gigantochloa parviflora*	NC050749
35	*Gigantochloa verticillata*	MN688203
36	*Melocalamus arrectus*	PV925758
37	*Melocalamus compactiflorus* var. *fimbriatus*	MK679793
38	*Melocalamus scandens*	PV976839
**Outgroup**		
1	*Arundinaria fargesii*	MZ905456
2	*Arundinaria gigantea*	JX235347

**Table 2:** Composition of *Dendrocalamus* Species in Yunnan Province.

Number	Taxon	Number	Taxon
1	*D. asper*	20	*D. menglongensis*
2	*D. bambusoides*	21	*D. pachycladus*
3	*D. barbatus* var. *barbatus*	22	*D. pachystachyus*
4	*D. barbatus* var. *internodiiradicatus*	23	*D. parishii*
5	*D. birmanicus*	24	*D. peculiaris*
6	*D. brandisii*	25	*D. puerensis*
7	*D. calostachyus*	26	*D. semiscandens*
8	*D. farinosus*	27	*D. sikkimensis*
9	*D. fugongensis*	28	*D. sinicus* var. *sinicus*
10	*D. giganteus*	29	*D. sinicus* var. *pachyloenus*
11	*D. hamitonii*	30	*D. tibeticus*
12	*D. jianshuiensis*	31	*D. tomentosus*
13	*D. jinghongensis*	32	*D. yunnanicus*
14	*D. latiflorus*	33	*D. atroviridis*
15	*D. membranaceus* f. *membranaceus*	34	*D. menghanensis*
16	*D. membranaceus* f. *fimbriligulatus*	35	*D. xishuangbannaensis*
17	*D. membranaceus* f. *pilosus*	36	*D. yingjiangensis*
18	*D. membranaceus* f. *striatus*	37	*D. wazibii*
19	*D. membranaceus* var. *microphyllum*	38	*D. turgidinodis*

**Table 3:** Morphological comparison between *D. turgidinodis* and related species.

Characters	*D. turgidinodis*	*D. hamiltonii*	*D. fugongensis*	*D. sinicus*
Culm diameter	13–16 cm	9–13 cm	10–15 cm	20–30 cm
Intranode	swollen	not swollen	not swollen	swollen
Culm leaf sheath	deciduous	deciduous	deciduous	tardily deciduous or persistent
Culm leaf sheath shoulder	little prominent, margin serrate	little prominent	absent	absent
Culm leaf ligule	ca. 5 mm tall, margins dentate and bearing short oral setae	ca. 1 mm tall, margins dentate	ca. 3 mm tall, margins dentate	ca. 6 mm tall, margins dentate
Culm blade	erect, glabrous	erect, procumbent setae adaxially	erect, with procumbent setae adaxially	nearly erect or a little open, with sparse pubescence abaxially and setae among veins adaxially
Dominant branches	1–3	1	1 or undeveloped	undeveloped
Foliage leaf	11–33 cm × 1.5–8 cm, glabrous	ca. 38 cm × 7 cm, glabrous	18–25 cm × 3–4.2 cm, with dense yellow-brown pubescence at the abaxial base	20–40 cm × 4–6.5 cm, pubescent on both surfaces or nearly glabrous
Foliage leaf ligule height	1–1.5 mm	1.5–2 mm	1.5–2 mm	1.5–2 mm
Foliage leaf auricle	absent	absent	tiny, oral setae 5–7 mm long	absent
Foliage leaf sheath	glabrous	procumbent setae	glabrous	pubescent

## Data Availability

The original contributions presented in this study are included in the article; further inquiries can be directed to the corresponding authors.
